# An etiologic prediction model incorporating biomarkers to predict the bladder cancer risk associated with occupational exposure to aromatic amines: a pilot study

**DOI:** 10.1186/s12995-017-0167-4

**Published:** 2017-08-08

**Authors:** Giuseppe Mastrangelo, Angela Carta, Cecilia Arici, Sofia Pavanello, Stefano Porru

**Affiliations:** 1 Department of Cardiac, Thoracic, and Vascular Sciences, Unit of Occupational Medicine, University of Padova, Via Giustiniani 2 -, 35128 Padova, Italy; 2 Department of Medical and Surgical Specialties, Radiological Sciences and Public Health, Section of Public Health and Human Sciences, University of Brescia, Brescia, Italy; 3 University Research Center “Integrated Models for Prevention and Protection in Environmental and Occupational Health”, University of Brescia, Brescia, Italy; 4 Department of Diagnostics and Public Health, Section of Occupational Health, University of Verona, Verona, Italy

**Keywords:** Urinary bladder neoplasms, Occupational exposure, Risk, Logistic models, ROC curve

## Abstract

**Background:**

No etiological prediction model incorporating biomarkers is available to predict bladder cancer risk associated with occupational exposure to aromatic amines.

**Methods:**

Cases were 199 bladder cancer patients. Clinical, laboratory and genetic data were predictors in logistic regression models (full and short) in which the dependent variable was 1 for 15 patients with aromatic amines related bladder cancer and 0 otherwise. The receiver operating characteristics approach was adopted; the area under the curve was used to evaluate discriminatory ability of models.

**Results:**

Area under the curve was 0.93 for the full model (including age, smoking and coffee habits, DNA adducts, 12 genotypes) and 0.86 for the short model (including smoking, DNA adducts, 3 genotypes). Using the “best cut-off” of predicted probability of a positive outcome, percentage of cases correctly classified was 92% (full model) against 75% (short model). Cancers classified as “positive outcome” are those to be referred for evaluation by an occupational physician for etiological diagnosis; these patients were 28 (full model) or 60 (short model). Using 3 genotypes instead of 12 can double the number of patients with suspect of aromatic amine related cancer, thus increasing costs of etiologic appraisal.

**Conclusions:**

Integrating clinical, laboratory and genetic factors, we developed the first etiologic prediction model for aromatic amine related bladder cancer. Discriminatory ability was excellent, particularly for the full model, allowing individualized predictions. Validation of our model in external populations is essential for practical use in the clinical setting.

## Background

Bladder cancer (BC) accounts for 5–10% of all malignancies among males in Europe and USA [1]. The most important risk factors are smoking, genetic susceptibility and occupational exposure [2].

An excess BC risk was identified since the early 1950s in the rubber industry and was associated to the use of b-naphthylamine [3]. Small excesses of BC risk have continued to be observed even in more recent studies of rubber workers published in the 1980s and 1990s [4]. Other aromatic amines (AAs) have been shown to be carcinogenic [5]. Nonetheless, occupational exposure to AAs has continued due to their industrial and commercial value [6]. AAs exposure can occur at lower extent in many other occupational settings. A systematic review of Italian studies estimated that 4 to 24% of BCs are attributable to occupational exposure [7]. BC cases effectively compensated by INAIL were 440 from 2000 to 2006, on average 63 per year [8]. Because in Italy incident cases of BC in 2006 were about 17,000/ year in males [9], the expected cases of occupational disease should be between 680 (17,000×0.04) and 4080 (17,000×0.24), and underreporting between 91% ((63/680) -1) and 98% ((63/4080) -1).

There is a strong genotoxic mechanism for carcinogenicity of several AAs, and multiple metabolic pathways as well as many polymorphic genes have been found to be implicated in the activation of AAs into DNA-reactive intermediates [10]. Over the last 20 years, numerous biomarkers have been investigated in workers exposed to AAs [6]. A key biomarker are the DNA adducts which are considered as the ‘biologically effective dose’ because they represent an integrated measure of carcinogen exposure, absorption, distribution, metabolism and DNA repair [11].

Few studies have combined clinical factors with blood and urinary biomarkers into risk profiles that can be used to predict the likelihood of etiological diagnosis of BC [12–16]. To the best of our knowledge, no study has tried to use biomarkers to predict the etiological diagnosis of occupational BC. The causal attribution to occupation usually relies on thorough occupational history collection, pertinent and documented risk assessment, availability of industrial hygiene measurements, appraisal and reference to evidence based literature data; to reach etiological diagnosis, the likely best option is to refer patients to occupational health specialists. An etiologic prediction model tool divides an initial population of BC cases into a smaller fraction of ‘positives’ that should be referred to an occupational health specialist for etiologic assessments, and a larger portion of ‘negatives’ that should no longer be considered. To be effective, an algorithm should increase the number of single BC cases receiving an etiologic ascertainment cases, therefore leading to a decrease of underreporting and under-compensation; eventually, such actions are beneficial for the individual, as well as for public health.

The aim of the present study was therefore to find a biomarker profile enabling to discriminate AA-related BC from non-AA-related BC and evaluate the algorithm with the approach of Receiver Operator Characteristic (ROC) curves, within the framework of a well-established case-control study on BC.

## Methods

### Study design and population

The present study includes the “cases” arm stemming from of an earlier hospital-based case-control study fully described in previous publications [17–20]. Inclusion criteria were being male, aged 20–80, Italian. Cases were all 199 newly diagnosed, histologically confirmed BC patients, admitted to the Urology Departments of two large hospitals from 1997 to 2000. Controls were all 214 non-neoplastic urological patients matched to cases by age (±5 years), period and hospital of admission. A written informed consent was obtained from each subject; the local Ethical Committee approved the study.

### Data collection

Peripheral blood lymphocytes (PBLs) were collected and automated DNA extraction was performed according to Extragen kit (Extragen BC by TALENT) [17]. Genotyping of glutathione S-transferase M1 (GSTM1) null, GSTT1 null, GSTP1 I105V, N-acetyltransferase 1 (NAT1) fast, NAT2 slow, cytochrome P450 1B1 (CYP1B1) V432 L, sulfotransferase 1A1 (SULT1A1) R213H, myeloperoxidase (MPO) G-463A, catechol-O-methyltransferase (COMT) V108 M, manganese superoxide dismutase (MnSOD) A-9 V, NAD(P)H:quinone oxidoreductase (NQO1) P187S, X-ray repair cross-complementing group 1 (XRCC1) R399Q, XRCC3 T241 M, and xeroderma pigmentosum complementation group (XPD) K751Q polymorphisms was assessed using Amplification Refractory Mutation System assay [17]. Bulky-DNA adducts were detected by 32P–postlabeling after Nuclease P1 enrichment and labelled adducts resolution on Thin Layer Chromatography (TLC) [20]. DNA adducts levels were measured as relative adduct level per 10^8^ nucleotides. A trained interviewer collected information on demographic variables, lifetime smoking history, coffee and other liquid consumption, dietary habits, lifetime occupation history by questionnaire. Job titles and individual activities, as well as occupational exposures to AAs, were blindly coded by an occupational physician according to methodology described in previous publication [21]. Occupations involving exposure to AAs were attributed to 11 International Standard Classification of Occupations (ISCO, International Labour Office, 1968) codes for job tasks (1–61.30: Painter, Artist; 3–70.20: Mail Sorting Clerk; 5–70.30: Barber-Hairdresser; 7–41.40: Mixing- and Blending-Machine Operator, Chemical and Related Processes; 8–01.10: Shoemaker, General; 8–11.20: Cabinetmaker; 8–73.70: Vehicle Sheet-Metal Worker; 9–01.35: Rubber Moulding-Press Operator; 9–31.20: Building Painter; 9–39.20: Brush-Painter, except Construction; 9–39.30: Spray-Painter, except Construction) and 11 International Standard Industrial Classification of all Economic Activities (ISIC, United Nations, 1968) codes for industrial activities (3240: Manufacture of footwear, except vulcanized or moulded rubber or plastic footwear; 3320: Manufacture of furniture and fixtures, except primarily of metal; 3521: Manufacture of paints, varnishes and lacquers; 3559: Manufacture of rubber products not elsewhere classified; 3819: Manufacture of fabricated metal products except machinery and equipment not elsewhere classified; 3824: Manufacture of special industrial machinery and equipment except metal- and wood-working machinery; 3843: Manufacture of motor vehicles; 5000: Construction; 9415: Authors, music composers and other independent artists not elsewhere classified; 9513: Repair of motor vehicles and motorcycles; 9591: Barber and beauty shops).

### Best-case definition

We investigated exposure to AA in all jobs held during lifetime, carefully assessing the level and the temporal aspects of such exposure according to standardized procedures [21]. Then, in order to achieve an optimal case definition [22], the critical values for time since first exposure (TSFE) and time since last exposure (TSLE) were chosen based on literature findings. In a cohort of Italian dyestuff workers [23], the risk of BC mortality decreased with increasing TSLE and became non-significant at ≥30 years since last exposure. Out of 19 BC patients observed in a Japanese dyestuff-plant, 17 showed a TSFE ≥20 years and 18 a TSLE ≤35 years [24]. Thus, the criteria for best-case definition were: TSFE higher than 20 years; TSLE lower than 35 years; length of exposure of at least 1 year; any value of cumulative exposure to AAs. The 15 BC cases complying with the above criteria were considered AA-related bladder cancer.

### Variables and statistical analyses

Smoking was a categorical variable with three levels: nonsmokers; former smokers from >20 years; current smokers and former smokers from less than 20 years. Life-long time-weighted average of cups/day of coffee and age at diagnosis were broken down in four classes according to the tertiles. DNA adducts were transformed in logarithm, and all values >1 were coded as 1 and otherwise as 0. Genetic biomarkers were coded as 0/1 variables as follows: *GSTM1* (“*NULL*” variant =1, otherwise = 0); *GSTP1* (“*1A/1A*” = 0, otherwise = 1); *GSTT1* (“*NULL*” = 1); *NAT1* (“*S*” = 1); *NAT2* (“*S*” = 1); *MPO* (“*A/A*” = 1); *COMT* (“*WW*” = 1); *MnSOD* (“*WW*” = 1); *NQO1* (“*MM*” = 1); *CYP1B1* (“*WW*” = 0); *XRCC1* (“*G/G*” = 0); *XRCC3* (“*C/C*” = 0); *XPD* (“*A/A*” = 0). All the above variables became the predictors in a model of logistic regression in which the dependent variable was 1 for the 15 patients (cases) with AA-related BC (see above) and 0 for the other 184 BC patients (controls). A stepwise selection of independent variables was made using 0.10 as “p-to-enter” and 0.15 as “p-to-remove”. Therefore, from the same sample of 199 cases of BC, two algorithms were obtained (full model and short model) reporting for each regressor the OR with 95% CI and *p*-value. The best fitting model was chosen with measures of predictive power (R-square and area under the ROC curve) and GOF statistics (Pearson chi-square and Hosmer-Lemeshow test). The criterion was “the higher the better” for the former, and “*p*-value above 0.05” for the latter. The graphical outputs of the ROC curves were obtained and the AUC was interpreted according to the classification proposed: 0.5 (not informative test); 0.5–0.70 (inaccurate test); 0.7–0.9 (moderately accurate test); 0.9–1.0 (highly accurate test); 1 (perfect diagnostic test) [25]. A statistical test comparing the equality of AUCs was also calculated. Lastly, using the prediction equation we obtained a new variable containing the model-predicted probability of a positive outcome; the same computer program provided the “best cut-off” of predicted probability [26] that maximized the difference between BC patients with or without AA-related disease in both models. Using such value we built the classification table (true positive, false positive, true negative and false negative) from which we calculated sensitivity, specificity, positive and negative predictive values and diagnostic accuracy of each model. Purely statistical measures for comparing two risk prediction models have, however, limited use for medical decision making because they do not incorporate harms and benefits related to treatment decisions arising from the risk prediction model [27]. To evaluate whether clinical use of prediction models, diagnostic tests, and molecular markers would do more good than harm, a simple type of decision analysis (net benefit, NB, approach) has been used [28]. The NB depends on the benefit B, the cost C, the prevalence P of the outcome, and the risk threshold, R, which expresses the model’s classification accuracy, that is the ability of the risk model to assign high risks to cases and low risks to controls. The key quantity is R, which is a function of the harms and benefits of the possible outcomes without detailed specification of harms and benefits [29]. Therefore, the NB to the population of using the risk model is:

$$ {NB}_R=\left({TPR}_R\times P\right)-\left(\left(\frac{R}{1-R}\right)\times {FPR}_R\times \left(1-P\right)\right) $$

where:

TPR_R_ = true positive rate, also called sensitivity;

FPR_R_ = false positive rate, also called one minus specificity;

P = probability of diseases at a given time, also called prevalence;

R = risk threshold, also called model-predicted probability of a positive outcome.

Net benefit can be plotted against a range of R, in what is called a “decision curve”. Decision curves are now widely used in the literature [28]. In the present study, however, wider effects on NB were observed with variations of P rather than of R.

### Sample size

In the present one-sample study, the sample size was estimated based on Fisher’s z test assuming a correlation 0.28 (that between DNA adducts and the 0/1 variable “presence/absence of occupational AA-related BC”) and a significance level of 0.05. For a two-sided hypothesis test, the estimated sample size was 98 or 130 patients setting the power at 0.80 or 0.90, respectively [30]. The actual number of BC patients was 199. All statistical analyses were performed with STATA 13.

## Results

Table 1 shows the main characteristics of BC patients and their distribution by genotypes (only patients with value set at 1 according to the above definitions). Those with disease related to AAs showed higher level of adducts, lower mean age (with higher percentage of youngest subjects) and higher number of smokers. Except for *COMT*, *GSTM1* and *GSTP1* other differences were insignificant.

**Table 1:** Occupational AA-related BC cases and other BC cases by DNA adducts, age, smoking categories, pack-years, coffee consumption and genotypes

Variables		15 AA-related BC cases		184 other BC cases	
		Mean (SD)	Number (%)	Mean (SD)	Number (%)
DNA adducts×10^8^ nucleotides (ln)		1.40 (1.5)		0.76 (1.2)	
	≥1		11 (73.3)		69 (37.5)
Age (years)		60.0 (10.4)		63.4 (11.2)	
	≤ 56.9 years		7 (46.7)		43 (23.4)
	57–65.9 years		3 (20.0)		50 (27.2)
	66–70.9 years		3 (20.0)		44 (23.9)
	≥ 71 years		2 (13.3)		47 (25.5)
Smoking (categories)					
	Non Smokers & Ex Smokers quitting >20 years		2 (13.3)		35 (19.0)
	Ex Smokers quitting <20 years		1 (6.7)		60 (32.6)
	Current smokers		12 (75.0)		87 (47.3)
Pack years (cigarettes smoked lifetime)		31.5 (12.3)		35.6 (26.2)	
	≤ 18.9		1 (6.7)		46 (25.1)
	19–32.9		8 (53.3)		45 (24.6)
	33–46.9		4 (26.7)		46 (24.6)
	≥ 47		2 (13.3)		47 (25.7)
Coffee consumption (weighted mean)		2.1 (1.7)		2.4 (2.4)	
	≤ 1 cup/day		6 (40.0)		71 (38.8)
	2 cups/day		4 (26.7)		36 (19.7)
	3 caps/day		3 (20.0)		32 (17.5)
	≥ 4 cups/day		2 (13.3)		44 (24.0)
Genotypes (legend below)	*GSTM1*		12 (80.0)		117 (63.9)
	*GSTT1*		3 (20.0)		38 (20.8)
	*GSTP*		5 (33.3)		92 (50.3)
	*NAT1*		4 (26.7)		60 (32.8)
	*NAT2*		10 (66.7)		111 (60.7)
	*MPO*		1 (6.7)		6 (3.3)
	*COMT*		7 (46.7)		132 (72.1)
	*MnSOD*		4 (26.7)		63 (34.4)
	*CYP1B1*		12 (80.0)		156 (82.3)
	*XRCC1*		7 (46.7)		98 (53.4)
	*XRCC3*		6 (40.0)		103 (56.3)
	*XPD*		7 (46.7)		113 (61.8)

*GSTM1* Glutathione S-transferase M1, *GSTT1* Glutathione S-transferase T1, *GSTP1* Glutathione S-transferase P1, *NAT1* N-acetyltransferase isozymes 1, *NAT2* N-acetyltransferase isozymes 2, *MPO* Myeloperoxidase, *MnSOD* Manganese Superoxide Dismutase, *COMT* Catechol-O-methyltransferase, *CYP1B1* Cytochrome p450 1B1, *XRCC1* X-ray repair cross-complementing protein 1, *XRCC3* X-ray repair cross-complementing protein 3, *XPD* xeroderma pigmentosum group D

Table 2 shows the logistic regression analysis obtained with the full and short models (with the subset of predictors chosen by the stepwise selection procedure). As expected from distribution of BC cases, significant ORs were few (DNA adducts and *COMT*) in both models. Nonetheless, measures of predictive power (R-squares and AUCs) were elevated. The full model seemed more performing, although both models passed the test and were correctly specified.

**Table 2:** Logistic regression (full and short models)

Variables	Classes	Full model			Short model		
		OR	95% CI	*p*-value	OR	95% CI	*p*-value
Age (years)^a^	57–65.9	0.12	0.01–0.98	0.048			
	66–70.9	0.29	0.04–2.25	0.239			
	≥ 71	0.10	0.01–1.07	0.056			
Smoking^b^	Ex-smokers	0.05	0.00–1.47	0.083	0.11	0.01–1.49	0.096
	Smokers	1.16	0.16–8.60	0.887	1.56	0.31–7.94	0.589
Coffee consumption^c^	2	0.63	0.09–4.64	0.654			
	3	1.96	0.27–14.24	0.505			
	≥4	0.05	0.00–0.86	0.039			
DNA adducts^d^	≥1	19.20	2.52–146.	0.004	6.02	1.66–21.8	0.006
Genotypes^e^	*GSTM1*	3.01	0.51–17.7	0.223			
	*GSTT1*	0.29	0.04–1.92	0.198			
	*GSTP1*	0.19	0.03–1.08	0.061	0.41	0.12–1.38	0.150
	*NAT1*	0.27	0.05–1.52	0.139			
	*NAT2*	2.55	0.52–12.6	0.25			
	*MPO*	1.38	0.03–65.1	0.871			
	*COMT*	0.05	0.01–0.39	0.005	0.21	0.06–0.72	0.014
	*MnSOD*	0.30	0.06–1.49	0.142			
	*CYP1B1*	0.27	0.03–2.42	0.24			
	*XRCC1*	0.97	0.22–4.22	0.966			
	*XRCC3*	0.38	0.09–1.68	0.203	0.40	0.12–1.34	0.139
	*XPD*	0.31	0.05–1.81	0.195			
Constant term		5.95	0.06–545.	0.439	0.18	0.03–1.30	0.09
Reference groups: ^a^patients with ≤56.9 years; ^b^non smokers and ex-smokers from >20 years; ^c^patients with consumption of ≤1 cup/day; ^d^patients with <1 (logarithm values) of DNA adducts×10^8^ nucleotides; ^e^patients with genotype values set at 0 (see text for definition).							
Measures of fit for logistic regression			Full model		Short model		
R-square			0.389		0.228		
Area under the ROC curve			0.931		0.856		
Pearson chi-square test:	*p*-value		0.087		0.826		
	no. covariates		197		44		
Hosmer-Lemeshow test:	*p*-value		0.130		0.942		
	no. groups		10		10		

**Fig. 1:**
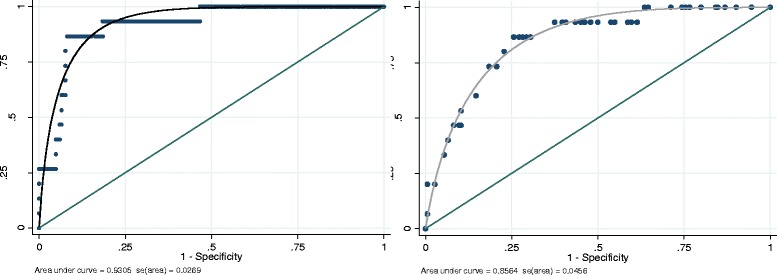
ROC curves obtained with the full model (*left*) and short model (*right*) of logistic regression; the AUC and its standard error is reported below the graph

Figure 1 shows the ROC curves obtained for the full and short model; the AUC and its standard error is also reported. A chi-square test comparing the two AUCs gave a value of 4.0438, with *p* = 0.0443, suggesting that including more variables (algorithm 1) could significantly improve the prediction (e.g. probabilities of occupational AA-related BC). The ROC area of model 1 (= 0.931) fell in the category of highly accurate tests; however, the cost of such high diagnostic accuracy was entering the algorithm 12 genotypes together with DNA adducts and some clinical factors.

Table 3 shows the 15 AA-related BC cases and 184 non-AA-related BC cases, classified as positive and negative according to the full or short model, always using the “best cut-off” of predicted probability provided by computer program to maximize the difference between BC patients with or without AA-related disease. With respect to the full model, the short one reduced the percentage of cases correctly classified (150/199 = 75% against 182/199 = 92%) by decreasing specificity (137/184 = 75% versus 169/184 = 92%). BC cases classified as ‘positive’ are those to be referred for evaluation by an occupational physician for etiological diagnosis; these patients were 28 (full model) or 60 (short model). Therefore, using 3 instead of 12 genotypes can double the number of patients to be referred for etiologic diagnosis. BC cases classified as ‘negative’ should be leaved out from etiologic workup. Since the negative predictive value was about 99% (169/171 according to the full model and 137/139 according to the short model), the model may be used to identify patients who can carefully avoid further evaluation. The other side of the coin was that two out of 15 true AA-related BC patients were classified as ‘false negative’ cases, which like the ‘true negatives’ should not undergo etiologic assessment of their disease. The NB per 100 patients was +4.9 and +4.1 in the left and right panel, respectively, when using panel-specific sensitivity, one minus specificity and the risk threshold R, along with the prevalence of AA-related BC, which was always 0.0754 (= 15/199). Comparing the left and right panel, values of NB were close in spite of divergent values of R.

**Table 3:** Classification of 15 AA-related BC cases (D) and 184 other BC cases (−D) according to the full model (left panel) or short model (right panel) of logistic regression

Classified	True		Total	Classified	True		Total
	D	–D			D	–D	
Positive	13	15	28	Positive	13	47	60
Negative	2	169	171	Negative	2	137	139
Total	15	184	199	Total	15	184	199
Sensitivity			86.7%	Sensitivity			86.7%
Specificity			91.9%	Specificity			74.5%
Positive predictive value			46.4%	Positive predictive value			21.7%
Negative predictive value			98.8%	Negative predictive value			98.6%
Correctly classified			91.5%	Correctly classified			75.4%
Net Benefit per 100 patients			+4.9	Net Benefit per 100 patients			+ 4.1

The risk threshold R (optimal cut-off point of predicted probability provided by the program) was 0.181 and 0.093 in the left and right panel, respectively. Net Benefit per 100 patients calculated from the above values

Table 4 shows the results of a different strategy in which we purposely reduced the “best cut-off” of predicted probability supplied by computer program in order to reduce the false negatives (1 in place of 2). The cost balancing the benefit (decrease of false negatives and increase of true positives) was a higher number of positives (from 28 to 48 with full algorithm; from 60 to 87 with short algorithm) requiring referral to an occupational physician. The NB per 100 patients was +5.3 and +5.1 in the left and right panel, respectively, when using panel-specific sensitivity, one minus specificity and the risk threshold R, along with the prevalence of AA-related BC, which was always 0.0754 (= 15/199). Comparing the left and right panel, values of NB were close in spite of the divergent values of R.

**Table 4:** Classification of 15 AA-related BC cases (D) and 184 other BC cases (−D) according to the full model (left panel) or short model (right panel) of logistic regression

Classified	True		Total	Classified	True		Total
	D	–D			D	–D	
Positive	14	34	48	Positive	14	73	87
Negative	1	150	151	Negative	1	111	112
Total	15	184	199	Total	15	184	199
Sensitivity			93.3%	Sensitivity			93.3%
Specificity			81.5%	Specificity			60.3%
Positive predictive value			29.2%	Positive predictive value			16.1%
Negative predictive value			99.3%	Negative predictive value			99.1%
Correctly classified			82.4%	Correctly classified			62.8%
Net Benefit per 100 patients			+ 5.3	Net Benefit per 100 patients			+ 5.1

The risk threshold R (purposely chosen cut-off point of predicted probability) was 0.09 and 0.05 in the left and right panel, respectively. Net Benefit per 100 patients calculated from the above values

**Fig. 2:**
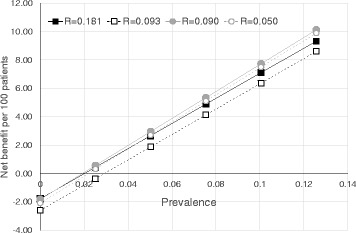
Decision curves of Net Benefit per 100 patients against different values of prevalence of AA-related BC, separately according to different values of risk threshold (R), i.e. cut-off point of predicted probability

The little effect on NB with variations of R is also shown in Fig. 2, which depicts NBs as squares and hollow squares (corresponding to R values of 0.181 and 0.093, respectively, see Table 3), or as circles and hollow circles (corresponding to R values of 0.090 and 0.050, respectively, see Table 4). By contrast wider differences and a steep increasing trend of NBs were found with prevalence of AA-related BC going from 0.0 to about 0.14. When prevalence approaches to 0.0, the sign of NBs became negative indicating costs that overwhelm benefits.

## Discussion

With the aim of finding a biomarker profile enabling to discriminate AA-related BC from non-AA-related BC, an etiologic prediction model was developed integrating 12 genotypes, DNA adducts, age, smoking and coffee consumption, while using 15 AA-related BC cases. The procedure classified the whole 199 BC patients in 28 positives and 171 negatives. The latter could be leaved out from etiologic workup, the former are those to be referred for etiological diagnosis. The cases correctly classified were 92% (182/199) and the discriminatory ability was excellent (AUC = 0.93). Examining 3 rather than 12 genotypes the cost of etiologic assessment increased because 60 (instead of 28) positives should receive a further testing (see Table 3, left and right panel). Nevertheless, there were two false negative cases. To overcome this detrimental outcome we used a second strategy (a lower risk threshold) that involved 1 false negative in place of 2 but increased the cost of diagnostic workup since the positives became 48 (instead of 28) or 87 (instead of 60) according to the full or short model, respectively (see Table 4, left and right panel). With the second strategy, despite the lower percentage of cases correctly classified and regardless of AUC reduction to values (0.7 to 0.9) indicating a moderately accurate test, the benefits were higher than the costs, as it can be seen by comparing the values of NB reported in the Tables 3 and 4.

In our earlier study [20], occupational AAs exposure was found to be positively associated with both BC risk (*p* = 0.041) and DNA adducts (*p* = 0.028). Since they were not associated with BC risk, DNA adducts were likely biomarkers of exposure. However, the responsible electrophilic substance could not be identified because adducts detected by the nuclease P1 method of 32P–post-labeling are not specific.

As shown in Table 2, many genotypes have given partial regression coefficients (namely, logarithms of ORs) that are not statistically significant. Each could be eliminated without significantly affecting the measures of fit for logistic regression, while the suppression of the whole set had a major effect. In fact, AUC of 0.931 for the full model reduced to 0.69 by entering DNA adducts as single predictor in the logistic regression. This occurs when the variables are strongly related to one another; if one is eliminated the other variables of the group act as substitutes. If, however, the entire group is removed do not remain other variables to compensate the lack of them [31]. In view of the above, we considered all regressors to obtain the predicted probability of a positive outcome, even though it can become difficult to attribute a meaning to each partial regression coefficient.

The etiologic prediction model tool that we have elaborated, enables to divide an initial population into a smaller fraction of “positives” and a larger portion of “negatives”. The former could be referred for further diagnostic assessments, the latter should be no longer considered. The “diagnosis” consists in attributing the disease to an exposure/occupational risk factor; this may happen or might be necessary in several context, such as individual case appraisal, compensation claims, litigation, health authority enquiries. Attributing the disease to an exposure/occupational risk factor may result in several advantages from clinical, epidemiological, individual and public health standpoints. Unfortunately, underreporting to health authorities and under compensation of occupational cancers are well known facts [32–34]. The latter evidence strengthens the need to adopt the second strategy aimed at increase as much as possible the identification of AA-related BC cases.

All BC patients are hospitalized at some point in time. Hospital physicians might then face two alternatives when dealing with a tumor, such as BC, with significant incidence and prevalence and with relevant attributable fraction of occupational risk factors: seeking for advice by an occupational health specialist for the patients or manage the case by themselves. A non-selective application of etiologic workup and appraisal would however results in a great loss of clinical and preventive opportunities. In addition to traditional methodology of etiological diagnosis, a reliable option could therefore be the tool described in this paper that enables discrimination of BC patients with high probability of occupational BC. However, attention should be paid to the underlying risk factors of AA-related bladder cancer. The greater the local degree of industrial development, the higher the chance of occurrence of an occupational disease, and the better the net benefit of using the tool that we have elaborated to ascertain this disease (see Fig. 2).

Validation of model in an external population is an essential next step towards practical use in the clinical setting. External validation requires a multicenter cohort and a prospective collection of data. At the end of the study, the individual characteristics of the validation cohort are multiplied by the regression coefficients of the corresponding variables (those obtained in the internal population) and the products are added to the constant term of the logistic regression. This value quantifies the individual predicted probability of having an AA-related BC. Subsequently, calibration plots are used to graphically explore the association between predicted probabilities and observed proportions: the points should be centered along a 45-degree line in the graph [12].

## Conclusions

BC cases with occupational AA-related disease can be individually assessed and stratified based on a predefined molecular biomarker profile. This tool can help ranking BC patients for referrals to an occupational physician for etiologic workup and appraisal. However, practical use in the clinical setting requires validation of the model in another population.

## Abbreviations

Aas: Aromatic Amines
BC: Bladder Cancer
COMT: Catechol-O-Methyltransferase
CYP1B1: Cytochrome P450 1B1
FPR_R_: False positive rate, also called one minus specificity
GSTM1: Glutathione S-Transferase M1 Null
GSTP1: Glutathione S-Transferase P1
GSTT1: Glutathione S-Transferase T1 Null
INAIL: Istituto Nazionale Assicurazione Infortuni Sul Lavoro
ISCO: International Standard Classification Of Occupations
Mnsod: Manganese Superoxide Dismutase
MPO: Myeloperoxidase
NAT1: N-Acetyltransferase 1
NB: Net Benefit
NQO1: NAD(P)H:Quinone Oxidoreductase
P: Probability of diseases at a given time, also called prevalence
Pbls: Peripheral Blood Lymphocytes
R: Risk threshold, also called model-predicted probability of a positive outcome
ROC: Receiver Operator Characteristic
SULT1A1: Sulfotransferase 1A1
TLC: Thin Layer Chromatography
TPR_R_: True positive rate, also called sensitivity
TSFE: Time Since First Exposure
TSLE: Time Since Last Exposure
XPD: Xeroderma Pigmentosum Complementation Group
XRCC1: X-Ray Repair Cross-Complementing Group 1
XRCC3: X-Ray Repair Cross-Complementing Group 3

## References

[1] Ferlay J, Parkin DM, Steliarova-Foucher E (2010). Estimates of cancer incidence and mortality in Europe in 2008. Eur J Cancer.

[2] Burger M, Catto JW, Dalbagni G, Grossman HB, Herr H, Karakiewicz P, Kassouf W, Kiemeney LA, La Vecchia C, Shariat S, Lotan Y (2013). Epidemiology and risk factors of urothelial bladder cancer. Eur Urol.

[3] IARC monographs on the evaluation of carcinogenic risk of chemicals to humans. The rubber industry. IARC Monogr Eval Carcinog Risk Chem Hum. 1982;28. http://monographs.iarc.fr/ENG/Monographs/vol1-42/mono28.pdf. Accessed 3 Nov 2015.

[4] Kogevinas M, Sala M, Boffetta P, Kazerouni N, Kromhout H, Hoar-Zahm S (1998). Cancer risk in the rubber industry: a review of the recent epidemiological evidence. Occup Environ Med.

[5] Baan R, Straif K, Grosse Y, Secretan B, El Ghissassi F, Bouvard V, Benbrahim-Tallaa L, Cogliano V (2008). WHO International Agency for Research on Cancer monograph working group, carcinogenicity of some aromatic amines, organic dyes, and related exposures. Lancet Oncol.

[6] Talaska G (2003). Aromatic amines and human urinary bladder cancer: exposure sources and epidemiology. J Environ Sci Health C Environ Carcinog Ecotoxicol Rev.

[7] Barone-Adesi F, Richiardi L, Merletti F (2005). Population attributable risk for occupational cancer in Italy. Int J Occup Environ Health.

[8] Scarselli A, Scano P, Marinaccio A, Iavicoli S (2009). Occupational cancer in Italy: evaluating the extent of compensated cases in the period 1994-2006. Am J Ind Med.

[9] AIRT Working Group (2006). Italian cancer figures -- report 2006: 1. Incidence, mortality and estimates. Epidemiol Prev.

[10] IARC monographs on the evaluation of carcinogenic risks to humans. Chemical agents and related occupations. IARC Monogr Eval Carcinog Risks Hum. 2012; 100F. http://monographs.iarc.fr/ENG/Monographs/vol100F/mono100F.pdf. Accessed .3 Nov 2015. PMC4781612 23189753

[11] Pavanello S, Pulliero A, Clonfero E (2008). Influence of *GSTM1 null* and low repair *XPC PAT+* on anti-B[a]PDE-DNA adduct in mononuclear white blood cells of subjects low exposed to PAHs through smoking and diet. Mutat Res.

[12] Lotan Y, Svatek RS, Krabbe LM, Xylinas E, Klatte T, Shariat SF. Prospective external validation of a bladder cancer detection model. J Urol. 2014;192:1343–8. 10.1016/j.juro.2014.05.087 24859442

[13] Kluth LA, Black PC, Bochner BH, Catto J, Lerner SP, Stenzl A, Sylvester R, Vickers AJ, Xylinas E, Shariat SF (2015). Prognostic and prediction tools in bladder cancer: a comprehensive review of the literature. Eur Urol.

[14] Terracciano D, Ferro M, Terreri S, Lucarelli G, D'Elia C, Musi G, de Cobelli O, Mirone V, Cimmino A (2017). Urinary long noncoding RNAs in nonmuscle-invasive bladder cancer: new architects in cancer prognostic biomarkers. Transl Res.

[15] Terreri S, Durso M, Colonna V, Romanelli A, Terracciano D, Ferro M, Perdonà S, Castaldo L, Febbraio F, de Nigris F, Cimmino A. New Cross-Talk Layer between Ultraconserved Non-Coding RNAs, MicroRNAs and Polycomb Protein YY1 in Bladder Cancer. Genes (Basel). 2016;7:127. 10.3390/genes7120127 PMC5192503 27983635

[16] Olivieri M, Ferro M, Terreri S, Durso M, Romanelli A, Avitabile C, De Cobelli O, Messere A, Bruzzese D, Vannini I, Marinelli L, Novellino E, Zhang W, Incoronato M, Ilardi G, Staibano S, Marra L, Franco R, Perdonà S, Terracciano D, Czerniak B, Liguori GL, Colonna V, Fabbri M, Febbraio F, Calin GA, Cimmino A. Long non-coding RNA containing ultraconserved genomic region 8 promotes bladder cancer tumorigenesis. Oncotarget. 2016;7:20636–54. 10.18632/oncotarget.7833 PMC4991481 26943042

[17] Shen M, Hung RJ, Brennan P, Malaveille C, Donato F, Placidi D, Carta A, Hautefeuille A, Boffetta P, Porru S (2003). Polymorphisms of the DNA repair genes *XRCC1*, *XRCC3*, *XPD*, interaction with environmental exposures, and bladder cancer risk in a case–control study in northern Italy. Cancer Epidemiol Biomark Prev.

[18] Covolo L, Placidi D, Gelatti U, Carta A, Scotto Di Carlo A, Lodetti P, Piccichè A, Orizio G, Campagna M, Arici C, Porru S (2008). Bladder cancer, GSTs, NAT1, NAT2, SULT1A1, XRCC1, *XRCC3*, *XPD* genetic polymorphisms and coffee consumption: a case-control study. Eur J Epidemiol.

[19] Pavanello S, Mastrangelo G, Placidi D, Campagna M, Pulliero A, Carta A, Arici C, Porru S (2010). CYP1A2 polymorphisms, occupational and environmental exposures and risk of bladder cancer. Eur J Epidemiol.

[20] Porru S, Pavanello S, Carta A, Arici C, Simeone C, Izzotti A, Mastrangelo G (2014). Complex relationships between occupation, environment, DNA adducts, genetic polymorphisms and bladder cancer in a case-control study using a structural equation modeling. PLoS One.

[21] Porru S, Placidi D, Carta A, Gelatti U, Ribero ML, Tagger A, Boffetta P, Donato F (2001). Primary liver cancer and occupation in men: a case-control study in high-incidence area in northern Italy. Int J Cancer.

[22] Coggon D, Martyn C, Palmer KT, Evanoff B (2005). Assessing case definitions in the absence of a diagnostic gold standard. Int J Epidemiol.

[23] Pira E, Piolatto G, Negri E, Romano C, Boffetta P, Lipworth L, McLaughlin JK, La Vecchia C (2010). Bladder cancer mortality of workers exposed to aromatic amines: a 58-year follow-up. J Natl Cancer Inst.

[24] Miyakawa M, Tachibana M, Miyakawa A, Yoshida K, Shimada N, Murai M, Kondo T (2001). Re-evaluation of the latent period of bladder cancer in dyestuff-plant workers in Japan. Int J Urol.

[25] Swets JA (1998). Measuring the accuracy of diagnostic systems. Science.

[26] Youden WJ (1950). Index for rating diagnostic tests. Cancer.

[27] Baker SG (2009). Putting risk prediction in perspective: relative utility curves. J Natl Cancer Inst.

[28] Vickers AJ, Calster BV, Steyerberg EW (2016). Net benefit approaches to the evaluation of prediction models, molecular markers, and diagnostic tests. BMJ.

[29] Kerr KF, Brown MD, Zhu K, Janes H (2016). Assessing the clinical impact of risk prediction models with decision curves: guidance for correct interpretation and appropriate use. J Clin Oncol.

[30] Demidenko E (2008). Sample size and optimal design for logistic regression with binary interaction. Stat Med.

[31] Armitage P (1971). Statistical methods in medical research.

[32] Fan ZJ, Bonauto DK, Foley MP, Silverstein BA (2006). Underreporting of work-related injury or illness to workers’ compensation: individual and industry factors. J Occup Environ Med.

[33] Straif K (2008). The burden of occupational cancer. Occup Environ Med.

[34] Eurogip. Reporting of occupational diseases: issues and good practices in five European countries. Ref. Eurogip-102/E February 2015. http://www.eurogip.fr/images/publications/2015/Report_DeclarationMP_EUROGIP_102EN.pdf. Accessed 3 Nov 2015.

